# Tetrahydropalmatine Alleviates Hyperlipidemia by Regulating Lipid Peroxidation, Endoplasmic Reticulum Stress, and Inflammasome Activation by Inhibiting the TLR4-NF-*κ*B Pathway

**DOI:** 10.1155/2021/6614985

**Published:** 2021-11-01

**Authors:** Ke Ding, Linjun Chen, Jiaqi He, Jiahong Wang, Chaohui Yu, Hui Wang

**Affiliations:** ^1^Traditional Chinese Medicine Dispensary, Zhejiang Provincial Hospital of Chinese Medicine, Hangzhou, Zhejiang 310000, China; ^2^General Surgery, Zhejiang Youth Hospital, Hangzhou, Zhejiang 310000, China

## Abstract

Hyperlipidemia (HLP) is a lipid metabolism disorder that can induce a series of cardiovascular and cerebrovascular diseases, such as atherosclerosis, myocardial infarction, coronary heart disease, and stroke, which seriously threaten human health. Tetrahydropalmatine (THP) is a component of the plant *Rhizoma corydalis* and has been shown to exert hepatoprotective and anti-inflammatory effects in HLP. However, whether THP regulates lipid peroxidation in hyperlipidemia, endoplasmic reticulum (ER) stress and inflammasome activation and even the underlying protective mechanism against HLP remain unclear. An animal model of HLP was established by feeding a high-fat diet to golden hamsters. Our results showed that THP reduced the body weight and adipose index; decreased the serum content of ALT, AST, TC, TG, and LDL-C; decreased the free fatty acid hepatic lipid content (liver index, TC, TG, and free fatty acid); inhibited oxidative stress and lipid peroxidation; extenuated hepatic steatosis; and inhibited ER stress and inflammasome activation in high-fat diet-fed golden hamsters. In addition, for the first time, the potential mechanism by which THP protects against HLP through the TLR4-NF-*κ*B signaling pathway was demonstrated. In conclusion, these data indicate that THP attenuates HLP through a variety of effects, including antioxidative stress, anti-ER stress, and anti-inflammatory effects. In addition, THP also inhibited the TLR4-NF-*κ*B signaling pathway in golden hamsters.

## 1. Introduction

Hyperlipidemia (HLP) is a disorder of abnormal lipid metabolism that manifests as one or several lipid disorders in plasma, including elevated plasma cholesterol, triacylglycerol, and low-density lipoprotein cholesterol; increased fat accumulation; or reduced high-density lipoprotein cholesterol levels [1]. HLP is the main cause of atherosclerosis and coronary heart disease, accounting for approximately 23.5% of all deaths worldwide [2]. Numerous studies have shown that the pathogenesis of HLP includes inflammatory factors, oxidative stress, endoplasmic reticulum stress, intestinal flora, and genetic polymorphisms [3, 4]. In recent years, with the rapid development of the economy and the impact of many factors, such as dietary habits with high cholesterol and lifestyle changes without exercise, the number of patients with HLP has increased dramatically [5]. The drugs used to treat HLP mainly include statins, fibrates, cholic acid chelating agents, and niacin [6–8]. Although these hypolipidemic drugs can effectively and rapidly reduce blood lipids, their long-term use can cause toxicity and side effects, such as abdominal distension, diarrhea, muscle weakness, muscle dissolution, and other adverse reactions [9]. Plant extracts are considered to be more suitable for long-term dietary supplementation due to their low toxicity and have the potential to be exploited as safe and effective lipid-lowering drugs.

Tetrahydropalmatine (THP) is an alkaloid extracted from the plant *Rhizoma corydalis* that has been proven to have anti-inflammatory [10], antioxidant [11], antiapoptotic [12, 13], lung protective [14], nerve protective [13], heart protective [15], and sedative and analgesic effects [16]. In addition, THP has been reported to regulate lipid metabolism. Guang et al. found that THP can inhibit lipid accumulation in 3T3-L1 adipocytes through the AMPK signaling pathway [17]. Recently, it was reported that THP exerted protective effects against HLP by inhibiting inflammation through modulating TLR4 and TRAF-6 in golden hamsters [18]. However, the specific mechanism in which it reduces HLP is still unclear.

Here, the model used was high-fat diet-induced HLP in golden hamsters. The present study was carried out to study the critical role of THP in lipid peroxidation, ER stress, and inflammasome activation involved in liver injury in high-fat diet-induced HLP and to unveil the possible mechanisms.

## 2. Materials and Methods

### 2.1. Animals and Chemicals

Sixty healthy golden male hamsters (100–120 g) of SPF grade were purchased from Beijing Weitong Lihua Experimental Animal Co., Ltd. (SCXK(Jing)2016–0011), and they were acclimatized and habituated to the laboratory for at least one week. All animals were housed separately and were provided free access to standard food and water in a controlled environment (temperature 22 ± 2°C and humidity 50 ± 10%) with 12 hours of light/darkness. Forty golden hamsters were selected for model preparation and fed a high-fat diet (containing 10% lard, 5% custard powder, 2% cholesterol, and 0.5% bile salt); another 8 were used as the normal control group and fed ordinary feed. High-fat diet-fed golden hamsters were randomly divided into 5 groups according to blood lipids and body weight, with 8 in each group, and maintained on the high-fat feed. The hamsters were sacrificed 6 weeks later, i.e., the 10th week of the experiment, for subsequent analysis. The protocol was approved by the Institutional Animal Care and Use Committee of Hangzhou Yingyang Biomedical Research and Development Center (SYXK (Zhe) 2020–0024). The THP (CAS: 6024-85-7, >98% purity) used in this study was purchased from Chengdu Gleep Biotechnology Co., Ltd. (Cheng Du, China). Atorvastatin (344423-98-9, >98% purity) was purchased from Sigma (St. Louis, MO, USA).

### 2.2. Experimental Design

The experimental design and doses used in this study were as follows: control group (NC) hamsters received standard chow diet for 8 weeks; model group (M) hamsters were maintained on a high-fat diet for 2 weeks; low-, middle-, and high-dose (LD, MD, and HD) hamsters were maintained on a high-fat diet and treated with a low dose (6.3 mg/kg/day), middle dose (12.6 mg/kg/day), or high dose (25.2 mg/kg/day) of THP, respectively, via intragastric gavage for another 6 weeks; positive control group (PC) hamsters were maintained on a high-fat diet and treated with atorvastatin (1.0 mg/kg/day) via intragastric gavage for another 6 weeks. The doses of THP and atorvastatin used in this study were based on previous literature studies [18, 19]. The weight of the hamsters was measured every two weeks. The hamsters were fasted overnight and sacrificed at the end of the experiment. The liver, perirenal fat, and epididymal fat were removed rapidly and washed thoroughly with saline. Blood and liver specimens were collected for biochemical index determination, western blot analysis, or histopathological examination. The liver index and adipose index were calculated using the following formulas: liver index = hepatic wet weight/body weight × 100%; adipose index = (epididymal fat + perirenal fat)/body weight × 100%.

### 2.3. Biochemical Analysis

Alanine aminotransferase (ALT), aspartate transaminase (AST), total serum cholesterol (TC), total triglyceride (TG), low-density lipoprotein cholesterol (LDL-C), free fatty acid (FFA), malondialdehyde (MDA), superoxide dismutase (SOD), glutathione (GSH), and catalase (CAT) activity were measured in accordance with the instructions of commercially available kits (Nanjing Jiancheng Bioengineering Institute, China). All data were measured using a microplate reader (BioTek Instruments, Inc., Winooski, VT, USA).

### 2.4. Histological Analysis

For hematoxylin and eosin (HE) staining, liver tissues were dissected and fixed in 4% paraformaldehyde solution, dehydrated with gradient ethanol solution, and embedded in paraffin wax. All tissues were cut into 5 *μ*m thick sections and stained with HE. For oil red O staining, the liver tissues were cut at 10 *μ*m thickness. Staining was conducted using an oil red O kit (Jiancheng, Nanjing, China) to detect lipid droplet accumulation in hepatocytes according to the manufacturer's instructions. All sections were evaluated under an optical microscope (Olympus, Tokyo, Japan) and photographed at final magnifications of ×200 and ×400.

### 2.5. RNA Extraction and Quantitative Real-Time PCR (qRT-PCR)

Total RNA was isolated from tissues by TRIzol extraction (Thermo Fisher Scientific, Rockford, IL, USA) according to the manufacturer's instructions. Reverse transcription was performed using a cDNA Reverse Transcription Kit (Thermo Fisher Scientific). qRT-PCR assays for mRNA expression were performed using an ABI 7500 Real-Time PCR system (Applied Biosystems). GAPDH was used as an internal control. The specific primers used for gene amplification in this study were as follows: CHOP, forward primer: 5′-GGAAACAGAGTGGTCATTCCC-3′, reverse primer: 5′-CTGCTTGAGCCGTTCATTCTC-3′; GRP78, forward primer: 5′- CCTGCGTCGGTGTGTTCAA-3′, reverse primer: 5′-ATCGCCAATCAGACGCTCC-3′; caspase 12, forward primer: 5′- ACAGCACATTCCTGGTGTTTATG-3′, reverse primer: 5′- CAGACTCTGGCAGTTACGGTTG-3′; GAPDH, forward primer: 5′- GCCAAAAGGGTCATCATCTC-3′, reverse primer: 5′-GTAGAGGCAGGGATGATGTTC-3′.

### 2.6. Western Blot

Total proteins were lysed in RIPA lysis buffer (Beyotime Biotechnology, Shanghai, China), and the concentration of the protein samples was measured using a bicinchoninic acid (BCA) protein assay kit (Beyotime). Proteins were subjected to SDS-PAGE and then electrophoretically transferred onto PVDF membranes. Membranes were incubated with primary antibodies overnight at 4°C after blocking in 5% nonfat milk in TBST buffer, which was then coincubated with horseradish peroxidase (HRP)-conjugated anti-mouse/anti-rabbit IgG (Thermo Fisher Scientific). Proteins were visualized using a detection system of enhanced chemiluminescence (ECL), and the bands were analyzed using BandScan ImageJ software. The primary antibodies used in this study were as follows: anti-CHOP (diluted 1 : 1000, ab11419, Abcam), anti-caspase 12 (diluted 1 : 1,000, ab62484, Abcam), anti-GRP78 (diluted 1 : 100, ab21685, Abcam), anti-NLRP3 (diluted 1 : 500, ab214185, Abcam), anti-ASC (diluted 1 : 1000, ab155970, Abcam), anti-pro-IL-1*β* (diluted 1 : 500, ab2105, Abcam), anti-IL-1*β* (diluted 1 : 1500, ab9722, Abcam), anti-p-NF-*κ*B (p-p65) (diluted 1 : 2000, ab86299, Abcam), anti-NF-kB p65 (diluted 1 : 300, ab19870, Abcam), anti-TLR4 (diluted 1 : 500, ab13556, Abcam), anti-MyD88 (diluted 1 : 1000, ab133739, Abcam), and anti-*β*-actin (diluted 1 : 1000, ab8226, Abcam). *β*-Actin was used as an internal control.

### 2.7. Enzyme-Linked Immunosorbent Assay (ELISA)

Serum levels of the proinflammatory cytokine IL-18 were measured by a commercially available ELISA kit (eBioscience, MA, USA). The procedure was performed according to the manufacturer's instructions (R&D Systems, Minneapolis, MN).

### 2.8. Statistical Analysis

The data were analyzed using GraphPad Prism (version 6.0, GraphPad software) and SPSS (version 21.0, IBM SPSS). Data from three repeated independent experiments are expressed as the mean ± standard deviation. Comparisons between two groups or among multiple groups were analyzed using Student's *t*-test or one-way analysis of variance (ANOVA) with Tukey's post hoc test. *P* < 0.05 was considered to indicate a statistically significant difference.

## 3. Results

### 3.1. Effects of THP on the Body Weight and Adipose Index of Golden Hamsters with HLP Induced by a High-Fat Diet

**Figure 1:**
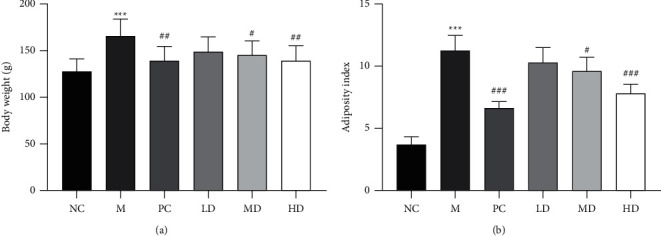
Effects of THP on the body weight and adipose index of golden hamsters with HLP induced by a high-fat diet. (a) Effect of THP on the body weight. (b) Effect of THP on the change in adiposity index. The results are expressed as the mean ± SD (*n* = 8). M versus NC, ^*∗∗∗*^*P* < 0.001; LD, MD, HD, and PC versus M ^#^*P* < 0.05, ^##^*P* < 0.01, and ^###^*P* < 0.001.

Consuming a high-fat diet for a long time can lead to abnormal accumulation of fat in the body, which can lead to weight gain [20, 21]. After continuous administration for 6 weeks, the weight and adipose index of hamsters in the M group were significantly higher than those in the NC group (Figures 1(a) and 1(b)). Compared with those in the M group, the weight and adipose index of hamsters in the PC, HD, and MD groups were significantly decreased, but there was no significant difference in the LD group (Figures 1(a) and 1(b)), indicating that THP has an antiobesity effect on fat formation induced by a high-fat diet.

### 3.2. Effects of THP on the Serum and Hepatic Lipid Contents of Golden Hamsters with HLP Induced by a High-Fat Diet

**Figure 2:**
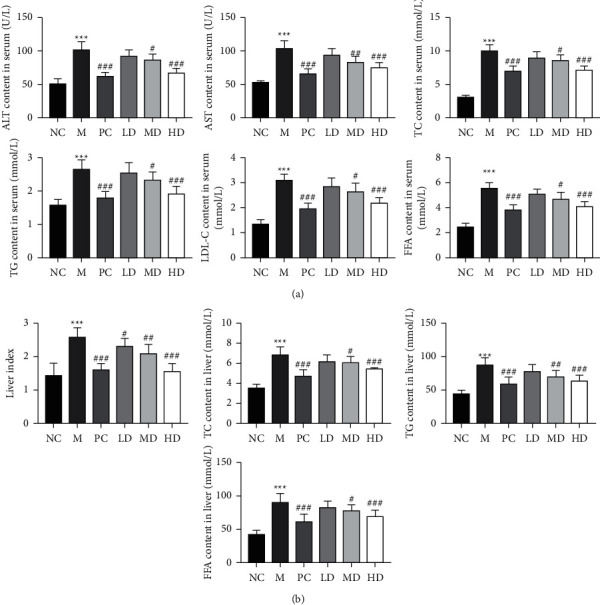
Effects of THP on the serum and hepatic lipid contents of golden hamsters with HLP induced by a high-fat diet. (a) Serum lipid contents of alanine aminotransferase (ALT), aspartate transaminase (AST), total serum cholesterol (TC), total triglyceride (TG), low-density lipoprotein cholesterol (LDL-C), and free fatty acid (FFA). (b) Liver lipid index, total serum cholesterol (TC), total triglyceride (TG), and free fatty acid (FFA). The results are expressed as the mean ± SD (*n* = 8). M versus NC, ^*∗∗∗*^*P* < 0.001; LD, MD, HD, and PC versus M ^#^*P* < 0.05, ^##^*P* < 0.01, and ^###^*P* < 0.001.

Next, we explored the effects of THP on the serum and hepatic lipid contents in golden hamsters fed a high-fat diet. Studies have confirmed that HLP is a disorder of lipid metabolism characterized by elevated levels of ALT, AST, TC, TG, LDL-C, and FFA [19, 22, 23]. Compared to the NC group, the serum contents of ALT, AST, TC, TG, LDL-C, and FFAs in the M group increased considerably after a high-fat diet (Figure 2(a)). Compared with the M group, the serum contents of ALT, AST, TC, TG, LDL-C, and FFA in the PC, HD, and MD groups were significantly decreased, but there was no significant difference in the LD group (Figure 2(a)). Moreover, the hepatic lipid contents (liver index, TC, TG, and FFA) of hamsters in the M group were significantly higher than those in the NC group, whereas treatment with different concentrations of THP (HD and MD groups) or atorvastatin (PC group) considerably decreased the elevation in the above parameters in the M group (Figure 2(b)). These results suggest that THP has lipid-lowering and protective effects against high-fat diet-induced liver injury.

### 3.3. Effects of THP on Oxidative Stress and Lipid Peroxidation in Golden Hamsters with HLP Induced by a High-Fat Diet

**Figure 3:**
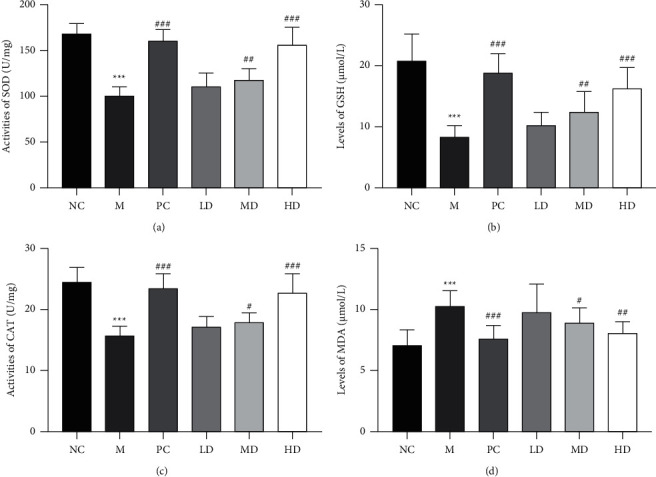
Effects of THP on oxidative stress and lipid peroxidation in golden hamsters with HLP induced by a high-fat diet. (a) Malondialdehyde (MDA) level, (b) superoxide dismutase (SOD) activity, (c) glutathione (GSH) level, and (d) catalase (CAT) activity. The results are expressed as the mean ± SD (*n* = 8). M versus NC, ^*∗∗∗*^*P* < 0.001; LD, MD, HD, and PC versus M ^#^*P* < 0.05, ^##^*P* < 0.01, and ^###^*P* < 0.001.

We also measured the antioxidant activity of THP in golden hamsters with HLP induced by a high-fat diet. Oxidative stress is a process of oxidative damage caused by the accumulation of reactive oxygen species (ROS) family members in the body or cells [24]. HLP causes the body to produce excessive ROS, reduces antioxidant enzyme activity, decreases the ability to scavenge free radicals, and causes changes in substances that reflect the level of oxidative stress, such as MDA, SOD, GSH, and CAT [25].  Figure 3 shows that the activities of SOD, GSH, and CAT, three key antioxidant enzymes, were decreased, while the activity of MDA, an indicator of lipid peroxidation, was considerably increased in the M group compared to the NC group. However, treatment with different concentrations of THP (HD and MD groups) increased the levels of SOD, GSH, and CAT and decreased the MDA level compared to the M group (Figures 3(a)–3(d)). These data indicate that THP exerts antioxidant activity in golden hamsters with HLP.

### 3.4. Effects of THP on Histological Changes in Liver Tissues of Golden Hamsters with HLP Induced by a High-Fat Diet

**Figure 4:**
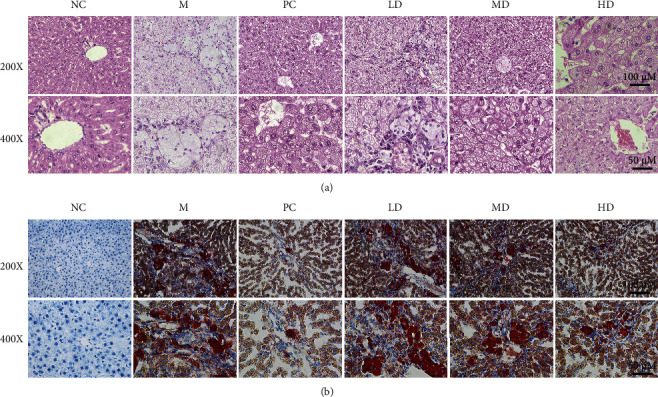
Effects of THP on the histological changes in liver tissues of golden hamsters with HLP induced by a high-fat diet. (a) Representative HE staining of golden hamster liver tissues in the NC, M, LD, MD, and HD groups. (b) Representative oil red O staining of golden hamster liver tissues in the NC, M, LD, MD, and HD groups (magnification: ×200, scale bar: 100 *μ*M; magnification: ×400, scale bar: 50 *μ*M).

To examine pathologic changes, we conducted HE and oil red O staining of livers. HE staining showed that the M group had mild hepatic steatosis; that is, lipid vesicles were seen in the liver (Figure 4(a)). After treatment with THP (MD and HD groups) or atorvastatin (PC group), the degree of hepatic steatosis improved, and lipid vesicles were significantly reduced (Figure 4(a)). Oil red O staining showed that HLP induced hepatic steatosis in the M group, and the hepatic lipid content was significantly reduced after treatment with THP (MD and HD groups) or atorvastatin (PC group) (Figure 4(b)). In the LD group, the degree of hepatic steatosis had little effect on lipid accumulation (Figures 4(a) and 4(b)).

### 3.5. Effects of THP on ER Stress and Inflammasome Activation in Golden Hamsters with HLP Induced by a High-Fat Diet

**Figure 5:**
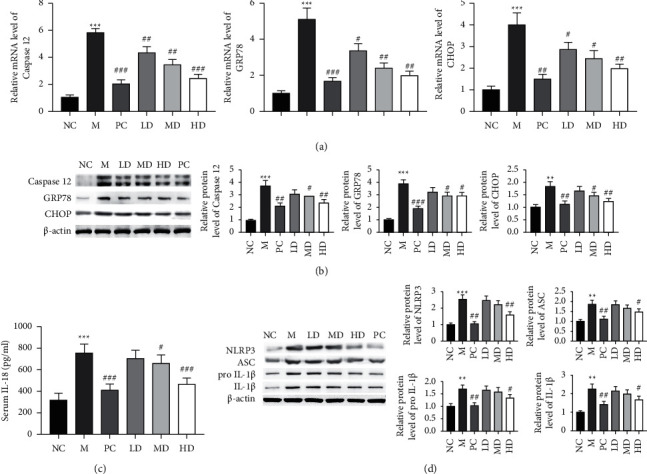
Effects of THP on ER stress and inflammasome activation in golden hamsters with HLP induced by a high-fat diet. (a) The mRNA levels of caspase 12, GRP78, and CHOP in liver tissues were evaluated by qRT-PCR. (b) The protein levels of caspase 12, GRP78, and CHOP in liver tissues were measured by western blotting, with *β*-actin as a loading control. (c) Serum IL-18 was determined by ELISA, and the results are expressed as the mean ± SD (*n* = 8). (d) The protein levels of NLRP3, ASC, pro-IL-1*β*, and IL-1*β* in liver tissues were measured by western blotting, with *β*-actin as a loading control. The results of at least three independent experiments are shown as the mean ± SD. M versus NC, ^*∗∗∗*^*P* < 0.001; LD, MD, HD, and PC versus (M) ^#^*P* < 0.05, ^##^*P* < 0.01, and ^###^*P* < 0.001.

HLP is usually accompanied by ER stress that leads to apoptosis and inflammation and further aggravates hepatocellular damage. As a member of the heat shock protein family, GRP78 plays a role as a molecular chaperone in the folding and assembly of newly synthesized proteins in the ER [26]. Abnormal expression of GRP78 can significantly aggravate cell damage caused by ER stress [27]. Prolonged or severe ER stress can cause apoptosis by activating several ER-specific proapoptotic factors, including CHOP and caspase 12 [28]. As shown in Figures 5(a) and 5(b), the mRNA and protein levels of CHOP, caspase 12, and GRP78 in the M group were increased compared to those in the NC group. However, treatment with THP (HD and MD groups) or atorvastatin (PC group) dramatically decreased the mRNA and protein levels of the above genes (Figures 5(a) and 5(b)). NLRP3 inflammasome activation is closely associated with the occurrence of some inflammatory diseases and metabolic abnormalities [29, 30]. The NLRP3 inflammasome is a multiprotein complex composed of the intracellular innate immune receptor NLRP3, the linker protein ASC, and the protease caspase 1 as the core, and the assembly of the complex can induce the maturation and secretion of the proinflammatory factors IL-1*β* and IL-18, thereby promoting inflammation [31, 32]. As shown in Figures 5(c) and 5(d), the protein expression of serum IL-18, NLRP3, ASC, pro-IL-1*β*, and IL-1*β* was upregulated in the M group, and the increase was inhibited by THP treatment (HD and MD groups) or atorvastatin treatment (PC group). However, there was little effect on the expression of the above genes in the LD group. These results suggest that the anti-inflammatory activity of THP included inhibition of ER stress and NLRP3 inflammasome activation.

### 3.6. Effects of THP on the TLR4-NF-*κ*B Signaling Pathway in Golden Hamsters with HLP Induced by a High-Fat Diet

**Figure 6:**
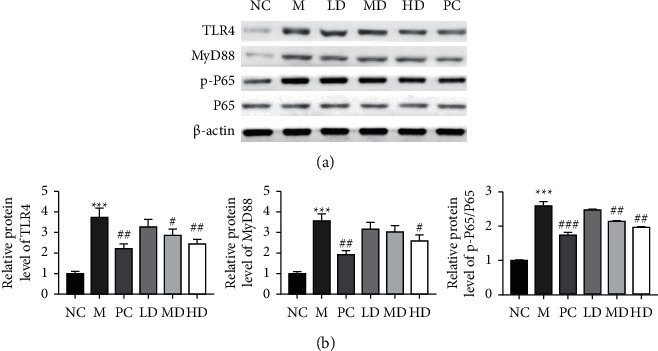
Effects of THP on the TLR4-NF-*κ*B signaling pathway of golden hamsters with HLP induced by a high-fat diet. (a) The protein levels of TLR4, MyD88, p-NF-*κ*B, and NF-*κ*B in liver tissues were measured by western blotting, with *β*-actin as a loading control. The results of at least three independent experiments are shown as the mean ± SD. M versus NC, ^*∗∗∗*^*P* < 0.001; LD, MD, HD, and PC versus M ^#^*P* < 0.05, ^##^*P* < 0.01, and ^###^*P* < 0.001.

There is increasing evidence that TLR4-NF-*κ*B is involved in metabolic abnormalities induced by a high-fat diet by regulating important processes, such as the inflammatory response, oxidative stress, and ER stress [33–39]. To reveal the potential mechanism of THP on golden hamster HLP and hepatic steatosis caused by a high-fat diet, the TLR4-NF-*κ*B signaling pathway was studied. The protein levels of TLR4, MyD88, and p-P65 were all upregulated in the M group compared to the NC group (Figures 6(a) and 6(b)). However, the protein levels of TLR4, MyD88, and p-P65 were significantly decreased by THP treatment (HD and MD groups) or atorvastatin treatment (PC group) compared to the M group, indicating that THP could regulate HLP induced by a high-fat diet in golden hamsters by suppressing TLR4-NF-*κ*B signaling.

## 4. Discussion

The liver is the most important location for the synthesis of lipids, and approximately 70 to 80% of the body's lipids are synthesized by the liver [40]. In the present study, golden hamsters were fed a high-fat diet to establish a hyperlipidemic model that demonstrates lipid metabolism similar to humans to serve as a good model for studying lipid metabolism disorders and for screening new lipid-lowering drugs [41]. THP improved obesity, lowered the serum and hepatic lipid contents, increased antioxidant activity, protected against liver injury, and inhibited ER stress and inflammation. Furthermore, the potential mechanism by which THP lowers lipids mediated by the TLR4-NF-*κ*B signaling pathway was also investigated.

Lipid metabolism disorder can cause liver adipose accumulation and damage liver function [42]. Apart from HLP caused by genetic inheritance, diet is one of the most important factors causing the disease. Long-term excessive consumption of foods that are high in fat, saturated fatty acids, and trans fatty acids can easily induce lipid accumulation, HLP, and NAFLD [21, 43]. In this study, THP considerably reduced the body weight and adipose index of golden hamsters. ALT and AST are important indicators of abnormal liver function. HLP may lead to abnormal levels of several blood lipid indicators (high TC, high TG, high LDL-C, high free fatty acid, etc.). THP attenuated the high-fat diet-induced increase in ALT, AST, TC, TG, LDL-C, and free fatty acid levels in serum and reduced the liver index, TC, TG, and free fatty acid levels in liver. These results were similar to those of Sun et al. [18]. In an increased lipid state, the generation of free radicals increases, which enhances lipid peroxidation and produces a large amount of lipid peroxides [44]. Yu et al. demonstrated that THP reduced intracellular ROS formation and the levels of MDA and LDH and increased the production of antioxidants (GSH and SOD) in human endothelial cells [11]. In a model of D-galactose-induced memory impairment in rats, THP reduced oxidative parameters (MDA and NO) and enhanced antioxidant parameters (SOD, CAT, and GSH) [45]. Consistent with the literature, we found for the first time that THP significantly increased the activities of SOD, CAT, and GSH and inhibited lipid peroxidation and MDA content in high-fat diet-induced HLP. Further histopathological staining results showed that THP alleviated liver steatosis and liver lipid content, providing strong evidence that THP alleviates HLP.

HFD can trigger systemic and hepatic inflammation and promote inflammasome activation [46, 47]. In this study, the increased level of IL-18 in serum induced by a high-fat diet was obviously decreased. Additionally, ER stress and the NLRP3 inflammasome exert an important role in metabolic diseases induced by a high-fat diet [30, 48, 49]. Our study is the first to discover the role of THP in inhibiting the activation of ER stress and the NLRP3 inflammasome in HLP, as evidenced by the reduced expression of CHOP, caspase 12, GRP78, NLRP3, ASC, pro-IL-1*β*, and IL-1*β*. TLR4 is the pattern recognition receptor most closely related to obesity in current research, and it is usually upregulated in animal models induced by a high-fat diet and is accompanied by enhanced downstream signaling pathways, such as NF-*κ*B signaling [50, 51]. Moreover, TLR4-NF-*κ*B can regulate the activation of NLRP3 inflammatory bodies and participate in various inflammatory diseases, such as hepatic lipid metabolism in obesity [52–54]. To investigate whether the effect of THP on HLP occurs through the regulation of TLR4-NF-*κ*B signaling, we evaluated the expression of TLR4, MyD88, p65, and p-P65. Activated MyD88 is a downstream effector of TLR4 that can recruit TRAF-6 and TGF-*β*-activated kinase 1 (TAK1) to transfer NF-*κ*B from the cytoplasm to the nucleus, resulting in the production of proinflammatory factors [55]. In this study, the protein levels of TLR4, MyD88, and p-P65 were considerably increased by a high-fat diet, and THP reduced the expression of the above proteins in a dose-dependent manner. These results suggest that TLR4-NF-*κ*B signaling may be involved in high-fat diet-induced HLP in golden hamsters.

## 5. Conclusion

In conclusion, the current study clearly demonstrates that THP can effectively alleviate HLP in high-fat diet-fed golden hamsters. THP inhibited lipid peroxidation, ER stress, and NLRP3 inflammasome activation, and the promising mechanism of THP is associated with inhibiting the TLR4-NF-*κ*B pathway. Overall, these data collectively suggest that THP may be a promising drug candidate for HLP, and more work should be done to elucidate the exact molecular mechanism of THP.

## Data Availability

All data generated or analyzed during this study are included in this published article.
